# Trends in the use of rehabilitation services in the community by people with mental disabilities in Israel; the factors involved

**DOI:** 10.1186/2045-4015-1-24

**Published:** 2012-06-20

**Authors:** Tzipi Hornik-Lurie, Nelly Zilber, Yaacov Lerner

**Affiliations:** 1 Falk Institute for Mental Health Research, Kfar Shaul Hospital, Givat Shaul, Jerusalem, 91060 Israel

**Keywords:** psychiatric rehabilitation, hostel, supported housing, vocational services, Rehabilitation of the Mentally Disabled Law

## Abstract

**Background:**

In 2001, the Rehabilitation of the Mentally Disabled Law was implemented, defining a basket of rehabilitation services to which people with mental disabilities are entitled.

**Objectives:**

To describe change over time in the characteristics of applicants to rehabilitation committees, types of referral agencies, and the proportion of those referred who were admitted. To identify factors affecting implementation of decisions to admit people with mental disabilities into different rehabilitation services and predictors of the length of time they remain in the services.

**Methods:**

The study population consisted of all applicants to rehabilitation committees during 2001-2008. Rehabilitation services included hostels, supported housing, and vocational services. Data were extracted from Ministry of Health rehabilitation and psychiatric hospitalization case registers. Findings were analyzed using descriptive statistics, Kaplan-Meier survival analyses, and Cox regressions.

**Results:**

There was a trend over time for more patients with shorter or no psychiatric hospitalization histories to be referred to rehabilitation services. Moreover, there was an increase in the proportion of referrals from the community, although the majority of referrals still came from psychiatric hospitals. Less than half of those recommended for a rehabilitation program were admitted and remained in a rehabilitation facility for one year or more. One factor predicting participants' longer stays in rehabilitation services after hospitalization was the proximity of the committees' decisions to the hospitalization. Another factor was the patient receiving vocational services while in residential care.

**Conclusion:**

Although over time the new law has resulted in a broader spectrum of people with mental disabilities receiving rehabilitation services, additional efforts are needed to enable them to remain in the system for a sufficient amount of time. Programs addressing specific needs should be developed accordingly.

## Background

During the last two decades a change has occurred in treatment of the mentally ill, putting increased emphasis on the development of rehabilitation services in the community [1-3]. According to this approach, the hospitalization period is intended for treatment of the acute phase of the mental illness and not for a prolonged stay. After the acute phase, efforts should be made to enable the disabled person to return as soon as possible to a normal and routine course of life and be integrated in the society [4-6]. All studies point to the fact that the wider the range of rehabilitation services provided to people with mental disabilities, the sooner and greater the improvement in their functioning [1,7,8].

In Israel also, as a consequence of collaborative efforts between social activists, mental health agents, and legislators, new legislation, the Rehabilitation of the Mentally Disabled in the Community Law of 2001 [9], was passed. From an international comparative perspective, this can be considered one of the most advanced laws in this area [10]. The Law defines a "basket" of rehabilitation services to which people with mental disabilities (at the level of 40% or more of psychiatric disability) until age 65 are entitled.

The "basket" provides a range of accommodations that reflect a continuum of support and staffing levels, from relatively low levels of support, such as supported housing with one to three participants in a rental apartment with staff visiting them on a regular basis, to high levels of support and staff, such as 24-hour-staffed facilities (hostels with about 20-30 tenants). Currently, about 60% of those in residential care are in supported housing vs. 40% in hostels.

Similarly, graded stepwise vocational services were developed in the community, from supported employment in the free market, through sheltered workshops where individuals work under protective conditions, to vocational clubs designed to teach vocational skills. Currently about 20% of those in vocational rehabilitation services are in supported employment in the free market, 30% are in sheltered workshops, and 50% in vocational clubs [11-13]. Those who are admitted to the rehabilitation services are entitled to accommodation and vocational rehabilitation simultaneously. "The basket of services attempts to address the key disadvantages consumers often face by providing them with services that focus on building skills and support in domains such as work, recreation, education, social life, and housing, to improve their opportunity to pursue valued social roles in the community." [14]. Regional rehabilitation committees, composed of a regional coordinator (usually a social worker), a psychiatrist, and a nurse, examine disabled persons and decide to which services the candidates are entitled. Candidates are referred by psychiatric hospitals, psychiatric outpatient clinics, and social agencies. The decisions of the committees are based on the recommendations of the referral agency and on the committee's assessment of the level of functioning and the severity of the symptoms of the candidates. The recommendations are valid for one year and are then required to be reviewed by a committee.

A number of articles (e.g., [13-15]) describe the law and its application. It has been shown that persons with severe mental illness who benefit from the psychiatric rehabilitation basket of services have better outcomes than those who do not [16]. Yet, according to the 2007 report of the State Comptroller [17], only about half of the people with mental disabilities for whom the committees recommended rehabilitation services ultimately utilized them. Although a decade has passed since the enactment of the legislation and the number of people having received the psychiatric rehabilitation "basket of services" had already reached 14,000 people in 2007 [12], there is no systematic study of the factors involved in the process of implementation of the decisions to admit people with mental disabilities into the rehabilitation services and the time they remain there [10].

The objectives of the present study were the following: 1. To describe the changes over the years in the characteristics of the applicants to the rehabilitation committees, the types of referral agencies, and the proportion of referred disabled persons admitted to the different rehabilitation services; and 2. To identify some of the factors affecting implementation of the decisions to admit people with mental disabilities into the different rehabilitation services and the predictors of the time they remain in these services.

## Methods

The study population consisted of all applications during 2001-2008 (excluding those for extensions) to the rehabilitation committees for at least one of the rehabilitation services (residential care or vocational rehabilitation). In 2007 the main part of the rehabilitation budget (80%) was allocated to residential care and vocational services, while home care services, social and leisure activity services, and supported education services drew only 20% of the budgetary resources [10]. This is the rationale for why the present study examined only the residential and vocational rehabilitation services, whether provided separately or concomitantly. Residential care can be defined hierarchically according to the degree and intensity of assistance and support provided to the residents by the staff [12]. Two types of residential care were thus defined: hostels, which serve users who need intensive 24-hour assistance and support, and supported housing, which serves users who are capable of living in the community with a relatively high degree of autonomy. Vocational rehabilitation services include vocational clubs, sheltered workshops, and supported employment [11]; no subdivision was performed.

Data (without any identifying information of the disabled persons) were extracted and merged from two registers of the Ministry of Health: the Rehabilitation Register and the Psychiatric Case Register.

The following independent variables were constructed:

• *Age at time of decision of the committee *- three categories: young adults (age 18-21 years), adults (age 22-55 years), older adults (age 56^+ ^years). A category of young adults was defined because some hostels are specifically designated for that age group.

• *Rehabilitation history *- six categories: being in a rehabilitation facility at the time of referral to a committee for a different facility (two categories), having been in one of the rehabilitation services in the past (three categories), no rehabilitation history.

• *Proximity of committee decision to a psychiatric hospitalization *- three categories: no hospitalization history, more than two months since release from previous psychiatric hospitalization, two months or less (It is most likely that the committee's decision will be affected by the hospitalization within this period), or within psychiatric hospitalization.

Two outcome variables were examined:

• *Time from the committee decision until its implementation *(admission to a rehabilitation unit);

• *Time in the rehabilitation program after admission*. A temporary interruption in the stay in a rehabilitation program of up to three months (usually because of a psychiatric hospitalization) was not considered as a termination of the participant's stay in rehabilitation, in keeping with the regulations of the Ministry of Health.

Descriptive statistics, Kaplan-Meier survival analyses, and Cox regressions were used to analyze the findings.

## Results

**Figure 1:**
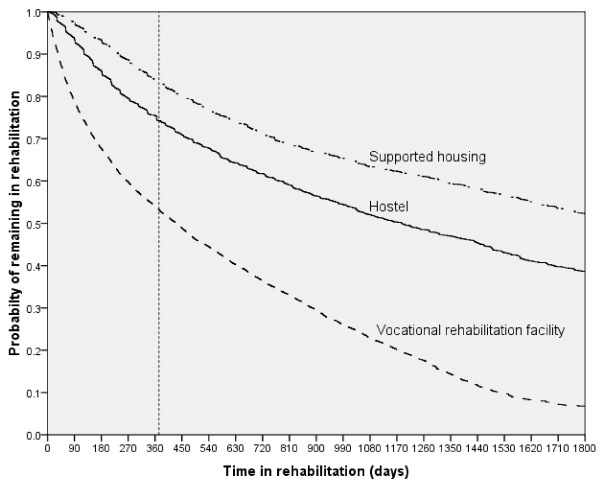
**Probability of remaining within the rehabilitation services by type of service over time (Kaplan-Meier survival analysis)**.

Approximately 19,000 applications met the criteria of our study, about half of which were for applicants new to the rehabilitation system. The other applications were for disabled persons who were temporarily released from the rehabilitation system because of a hospitalization of more than three months, for those who dropped out from the system because of other reasons, or who were recommended to change facility (e.g., from hostel to supported housing) or to add an additional service (e.g., addition of vocational rehabilitation to residential care). The applications were for hostels only (17%), for hostel and vocational rehabilitation (11%), for supporting housing only (16%), for supporting housing and vocational rehabilitation (14%), or for vocational rehabilitation only (42%). Most of the applications to the rehabilitation committees were approved (80-90%), but only 50-70% of the positive decisions were implemented within a year. The probability of remaining at least one year in a hostel was 0.75, in supported housing 0.85, and in vocational rehabilitation services 0.55 (Figure 1). In other words, less than half of those for whom the committees recommended rehabilitation were admitted to a rehabilitation facility and also remained in it for one year or more.

**Figure 2:**
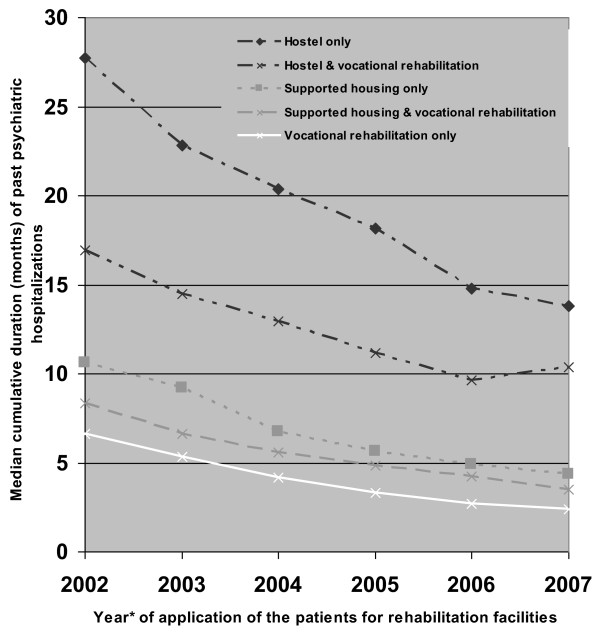
**Cumulative duration of past psychiatric hospitalizations of the applicants to a rehabilitation committee by year of the application and type of service requested**. *Every point represents the median of 3 sequential years.

**Figure 3:**
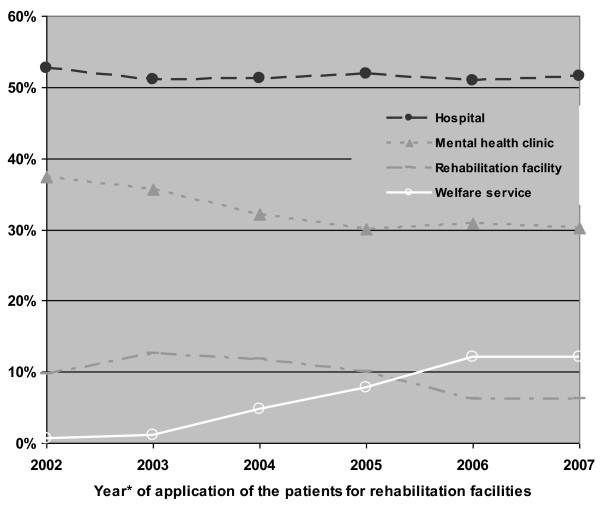
**Distribution of applications to residential care by referring agent and year of application (percent)**. *Every point represents the median of 3 sequential years.

**Figure 4:**
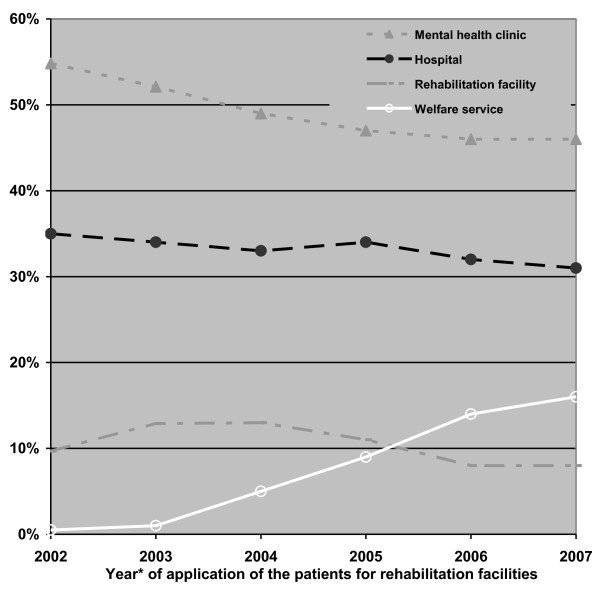
**Distribution of applications to vocational rehabilitation by referring agent and year of application (percent)**. *Every point represents the median of 3 sequential years.

Over the years, applications to the rehabilitation committees were of patients characterized by a shorter psychiatric hospitalization history or the absence of any former hospitalization. This trend could be observed for clients requesting any of the services, with patients requesting hostels experiencing the largest decrease in the median cumulative duration of past hospitalization (from 28 to 14 months) (Figure 2). Most of the referrals to the rehabilitation committees were from medical services. Concerning referrals to residential care, about 50% of the referrals were from hospitals and 33% from mental health clinics; of the referrals to vocational rehabilitation, about 33% were from hospitals and 50% from mental health clinics. On the other hand, the referrals from the welfare services, which were negligible during the first years, increased significantly by the end of the follow-up period to about 12% for residential care and about 16% for vocational rehabilitation (Figures 3 and 4).

**Figure 5:**
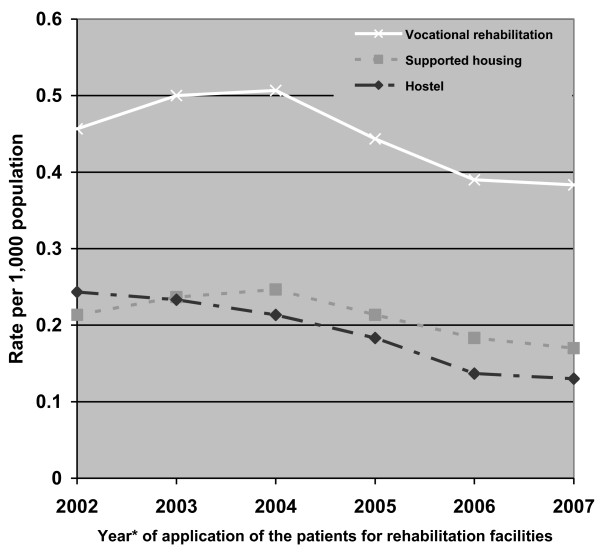
**Rates of application to a rehabilitation committee by year of the application and type of service requested (applications per thousand general population)**. *Every point represents the median of 3 sequential years.

The rates of application for both residential care (supportive housing and hostels) and vocational rehabilitation have tended to decrease since 2004. In addition, from that year, the rate of applications for supportive housing exceeded that of applications for hostels. Interestingly, this was true even among the applicants without a rehabilitation history, among whom the proportion of referrals to supportive housing increased over time, from 44% to 59%. This shift had already started during 2003-2004. The application for vocational rehabilitation services contributed the bulk of the applications during all the study years (Figure 5).

**Figure 6:**
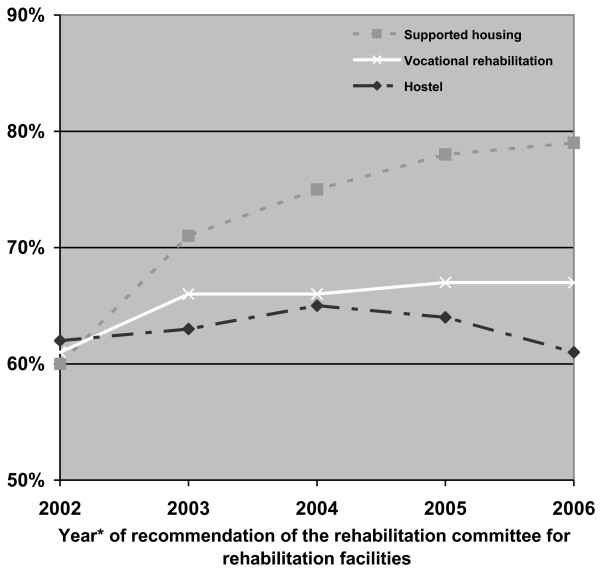
**Percentage of patients with implementation within one year from the recommendation of the rehabilitation committee, by type of rehabilitation services and year of recommendation**. *Every point represents the median of 3 sequential years. A follow up of one year was available only till 2007.

Over the years the proportion of disabled persons admitted to hostels within one year from the decision of the rehabilitation committees remained stable (about 62%), while the percentage of those admitted within one year to supported housing increased considerably, from 60% to 79%, and the percentage for those admitted to vocational rehabilitation increased slightly but still significantly, from 61% to 67% (Figure 6).

Two Cox analyses were performed, one to identify predictors of the time from the committee decision to its implementation and the second to identify predictors of the time the disabled persons remain in the three different types of rehabilitation services. In these analyses the variables examined were: age, rehabilitation history of the referees, and a service-related variable, proximity of committee decision to psychiatric hospitalization. In both analyses, the referring agency and regional committee were controlled for, and, for the second analysis, time from the committee decision to its implementation in rehabilitation services was also controlled for (Tables 1, 2 and 3). The key findings were as follows:

**Table 1:** Predictors of time from committee decision to admission and of time patients remain in hostel.

Independent variable	Prediction of time from committees' decision to its implementation^1^				Prediction of time patients remain in hostel^2^			
	N	OR	C.I. (95%)	*p*	N	OR	C.I. (95%)	*p*
**Age (years)**				0.000				0.000
18-21	477	0.67	0.59-0.78	0.000	263	1.63	1.38-1.90	0.000
22-55	3700	1.00			2672	1.00		
56+	465	1.01	0.89-1.14	0.926	326	0.81	0.69-0.95	0.010
**Rehabilitation history**				0.000				0.028
No rehabilitation history	2534	1.00			1706	1.00		
Hostel in the past	696	1.27	1.15-1.41	0.000	567	1.14	1.00-1.29	0.054
Supported housing in the past	75	1.13	0.85-1.50	0.405	53	1.41	0.97-2.05	0.068
Vocational or other rehabilitation in the past	678	1.04	0.93-1.16	0.499	481	1.07	0.93-1.22	0.333
Supported housing in the present	175	1.30	1.08-1.60	0.007	128	1.30	1.01-1.68	0.042
Vocational or other rehabilitation in the present	484	1.05	0.92-1.19	0.510	326	1.21	1.03-1.42	0.018
**Proximity of committees' decision to hospitalization**				0.000				0.586
No hospitalization history	279	0.73	0.61-0.87	0.001	158	1.11	0.89-1.38	0.365
More than two months since release from hospitalization	1238	0.66	0.60-0.73	0.000	765	0.98	0.87-1.11	0.799
Two months or less or within hospitalization	3125	1.00			2338	1.00		
**Total**	4642				3261			

^1^Controlling for referring agency and for regional committee. The higher OR is, the shorter the time from the committee decision to implementation.

^2^Controlling for: referring agency, regional committee, and time from the committees' decision to its implementation. The lower OR is, the longer the time the patients remain in the hostel.

**Table 2:** Predictors of time from committee decision to admission and time patients remain in supported housing.

Independent variable	Prediction of time from committees' decision to its implementation^1^				Prediction of time patients remain in supported housing^2^			
	N	OR	C.I. (95%)	*p*	N	OR	C.I. (95%)	*p*
**Age (years)**				0.024				0.000
18-21	222	0.91	0.77-1.07	0.260	168	2.24	1.82-2.77	0.000
22-55	4062	1.00			3133	1.00		
56+	472	1.15	1.02-1.28	0.017	367	0.90	0.76-1.08	0.264
**Rehabilitation history**				0.001				0.000
No rehabilitation history	2699	1.00			2015	1.00		
Supported housing in the past	309	0.96	0.83-1.11	0.600	257	0.87	0.70-1.09	0.231
Hostel in the past	86	0.94	0.73-1.22	0.654	69	2.16	1.63-2.87	0.000
Vocational or other rehabilitation in the past	639	1.01	0.91-1.26	0.784	496	1.00	0.86-1.16	0.991
Hostel in the present	207	0.65	0.54-0.79	0.000	172	1.09	0.83-1.42	0.543
Vocational or other rehabilitation in the present	816	0.99	0.90-1.10	0.963	695	0.86	0.74-0.99	0.036
**Proximity of committees' decision to hospitalization**				0.093				0.000
No hospitalization history	691	0.95	0.85-1.07	0.380	533	0.73	0.61-0.87	0.000
More than two months since release from hospitalization	2445	0.91	0.84-0.99	0.030	1885	0.79	0.70-0.90	0.000
Two months or less or within hospitalization	1620	1.00			1250	1.00		
**Total**	4756				3668			

^1^Controlling for referring agency and for regional committee. The higher OR is, the shorter the time from the committee decision to its implementation.

^2^Controlling for: referring agency, regional committee, and time from the committee decision to its implementation. The lower OR is, the longer the time the patients remain in the supported housing.

**Table 3:** Predictors of time from committee decision to admission and time patients remain in vocational rehabilitation.

Independent variable	Prediction of time from committees' decision to its implementation^1^				Prediction of time patients remain in vocational rehabilitation^2^			
	N	OR	C.I. (95%)	*p*	N	OR	C.I. (95%)	*p*
**Age (years)**				0.020				0.011
18-21	902	1.10	1.01-1.20	0.031	708	1.12	1.01-1.23	0.023
22-55	9724	1.00			7289	1.00		
56+	856	0.93	0.85-1.02	0.110	573	1.12	1.01-1.24	0.031
**Rehabilitation history**				0.000				0.008
No rehabilitation history	7082	1.00			5068	1.00		
Vocational in the past	2086	1.30	1.23-1.39	0.000	1701	1.02	0.95-1.09	0.569
Hostel and/or supported housing in the past	190	0.88	0.73-1.08	0.212	137	1.24	1.03-1.51	0.026
Other rehabilitation in the past	762	0.98	0.89-1.08	0.705	577	1.04	0.94-1.16	0.445
Hostel and/or supported housing in the present	404	0.87	0.76-1.00	0.050	336	0.84	0.73-0.97	0.016
Other rehabilitation in the present	958	1.10	1.01-1.20	0.024	751	0.92	0.84-1.02	0.113
**Proximity of committees' decision to hospitalization**				0.006				0.915
No hospitalization history	1919	1.12	1.03-1.21	0.006	1409	0.98	0.90-1.07	0.687
More than two months since release from hospitalization	6094	1.10	1.03-1.17	0.003	4655	0.99	0.93-1.06	0.744
Two months or less or within hospitalization	3469	1.00			2506	1.00		
**Total**	11482				8570			

^1^Controlling for referring agency and for regional committee. The higher OR is, the shorter the time from the committees' decision to its implementation.

^2^Controlling for: referring agency, regional committee, and time from the committee decision to its implementation. The lower OR is, the longer the time the patients remain in the vocational rehabilitation.

Hostel (Table 1):

• The young adults take more time to be admitted and they have a greater probability of leaving the hostel than the other age groups, especially the older adults.

• Those who are, or have been, in the rehabilitation system tend to be admitted to a hostel more rapidly than those without a rehabilitation history; but on the other hand, the latter group tends to remain in the system longer.

• Those who are referred to the rehabilitation committees during or close in time to a psychiatric hospitalization are admitted to a hostel significantly more quickly than the others, whether they were hospitalized previously or had no hospitalization history. The time patients remain in a hostel was not related to the proximity of the committee's decision for hospitalization.

Supported housing (Table 2):

• The older adults take less time to be admitted and the young adults have a greater probability of leaving than the other age groups.

• Those who are referred to supported housing while staying in a hostel are admitted after a significantly longer time than the others. As for duration of stay in supported housing, those applicants who had been in a hostel in the past have a significantly shorter stay.

• Among those with a psychiatric hospitalization history, the proximity of committee decision to a psychiatric hospitalization (two months or less) is significantly correlated with a shorter time until admission to supported housing, but with a shorter stay in the supported housing.

Vocational rehabilitation (Table 3):

• The young adults take less time to be admitted, and adults aged 22-55 tend to leave more quickly than the younger or older adults.

• Those who are referred to vocational rehabilitation and have a vocational rehabilitation history are admitted significantly more quickly than the others. Those who are staying in residential care have a better chance of remaining in the vocational rehabilitation program.

• The more distant the referral to the committee is from a psychiatric hospitalization, the sooner the applicant will be admitted to a vocational rehabilitation program, but the probability of remaining in the program is not affected by the proximity of the committees' decisions for psychiatric hospitalization.

## Discussion

A limitation of the study is that, although the rehabilitation register contains data on a nation-wide basis about the range and duration of the services provided to each individual, it lacks important personal information, mainly about the severity of the disease and of the disability, the degree of motivation for rehabilitation, the level of functioning, and the quality of life of the participants, as well the amount of available support from the family, Thus only a limited list of variables extracted from the case register could be examined.

The strength of the study is its nationwide extent and its longitudinal nature. Trends over the years since the law was implemented in 2001 could thus be analyzed. Before the law was implemented, only 4,600 persons participated in any rehabilitation program. After implementation of the law the number gradually increased, reaching about 16,000 in 2008.

This is consistent with the fact that the annual number of hospitalized psychiatric patients increased only from 13,000 to 14,000, although the number of people with mental disabilities eligible for rehabilitation services increased from 44,000 to 59,000 between 2001 and 2007.

During the first years, the main objective was to reduce the number of beds of the chronic mentally ill patients with a long psychiatric hospitalization by releasing them from the hospitals to the community [10]. This indeed resulted in a decrease of about 50% in the number of long-stay (more than one year) patients [12]. Over the years, an increasing proportion of new chronic patients were found to be referred (those with a shorter psychiatric hospitalization history or even with no prior hospitalization). Beginning in 2003, candidates, who previously were referred mainly by psychiatric hospitals or clinics, began to be referred in increasing numbers by welfare services. The referral of less severely disabled persons could also explain why over the years the rate of application for supportive housing began to exceed that for hostels. Indeed, the percentage of new applicants referred directly to supported housing without having previously been in a more protected residential facility increased over the years, exceeding since 2003 the percentage referred to a hostel. This trend will probably continue in the future. The topic of differential housing services for disabled persons at different levels of functioning has attracted much interest lately [18,19].

Interestingly, although the total number of residents in both hostels and supported housing increased over the years [15], the percentage of those recommended for supported housing who were admitted there increased significantly, while this was not true for hostels.

When discussing the length of stay in rehabilitation, we assumed that in general the longer the support of the rehabilitation services, the better the outcome. As the majority of the clients in the present study have a history of past hospitalization (90% of those admitted to residential care), most of them need supportive residential care for a long period, otherwise they will have a greater probability of being rehospitalized [20]. Leaving the vocational rehabilitation program early most likely represents a premature termination of the rehabilitation process and not further progress, considering the fact that the vocational rehabilitation services in Israel also include supported employment in the free market (about 20% of the participants). Recently (in 2010), the reason for leaving rehabilitation programs was added to the records of the Rehabilitation Register. Preliminary findings point to the fact that only a negligible percentage of them left the rehabilitation services because they reached sufficient independence (Ministry of Health, personal communication).

In the present study, half of the applications were for disabled persons who had already been in the rehabilitation system and were referred again, apparently because they had been hospitalized or had dropped out of the system. They were readmitted to the rehabilitation system more rapidly but, on the other hand, they remained in the system for less time than the new referrals, probably because candidates who dropped out once from the system are less suitable for the existing services. They possibly need more intensive support.

An important finding is that the probability of not remaining in any of the rehabilitation services is relatively high in the first year. A similar trend has been found by Struch et al. in Israel [15], as well as by Mueser et al. in the United States [21]. It is therefore worthwhile to invest special effort in the successful integration of people with mental disabilities in their first year in rehabilitation.

The young adult age group took more time to be admitted to residential care than the other age groups and remained for less time after being admitted. It seems that separate and more specifically tailored units for youngsters should be established, both for residential care and vocational rehabilitation, where special programs suitable for them should be planned.

The closer the proximity of the committee's decision to the release of the patient from psychiatric hospitalization, the sooner the patients are admitted to hostels or to supportive housing, This could be explained by the necessity of the hospital authorities to release patients [22] and thus they may put pressure on the rehabilitation services and even assist them in locating a residential facility. Admission to supportive housing close to hospitalization leads, however, to a shorter stay than those admitted a longer time after hospitalization. This is not the case for admission to hostels after hospitalization. It is therefore possible that admission to residential care after hospitalization should be graduated, not starting immediately in supported housing but rather in the more protective hostels, as also suggested in the literature [5,23]. This finding may have important implications for the future policy of de-institutionalization.

There is a tendency to wait at least two months before admitting patients released from psychiatric hospitalization to a vocational program, although there is no evidence that participants remain for a longer time in vocational rehabilitation when admission is delayed. On the other hand, placing the clients concomitantly in a residential facility does allow them to stay longer in vocational programs, probably because such residential facilities serve as a support for these participants.

Another issue that is of interest is why only 60-70% of the positive recommendations by the rehabilitation committees for a rehabilitation program are followed. Possible factors such as discrepancies between regions in the availability of rehabilitation services, lack of case managers, and lack of programs designed for specific groups should be examined.

The study was not a full analysis of the extent to which the rehabilitation program has succeeded. A study based on case registers cannot replace a follow up based on a direct examination of the progress of the participants. Such a direct examination is extremely important and should utilize face-to-face interviews with a focus on changes in functioning, quality of life, and realization of personal goals. In addition, case register data contain critical information for an individual follow-up of the sequential transitions between the different levels of residential care and vocational programs. These data enable us to identify points of no further progress and whether they are client-related or service-related, as also suggested by Aviram [10]. This will be the purpose of a future research study.

## Conclusion

Over the years the rehabilitation services have been reaching clients with a shorter hospitalization history, and an increasing proportion of candidates are referred from the community, but effort is still needed to help them remain in the system for sufficient time. Possible conditions to achieve this purpose should be explored and programs for specific needs should be developed.

## Competing interests

The authors declare that they have no competing interests.

## Authors' contributions

All three authors were involved in all aspects of the study. All authors read and approved the final manuscript.

## Authors' information

Tzipi Hornik-Lurie is a Ph.D. candidate at Ben-Gurion University of the Negev. She specializes in quantitative analysis in the field of the sociology of health. She is presently a researcher at the Falk Institute for Mental Health Research, Jerusalem, Israel.

Nelly Zilber, D. ès Sc., is a former member of the French National Center for Scientific Research. She is a neuro-epidemiology and psychiatric epidemiology specialist. She is presently a senior researcher at the Falk Institute for Mental Health Research, Jerusalem, Israel.

Yaacov Lerner MD, psychiatrist, is the former director of the Jerusalem Mental Health Center. He is a specialist in mental health service research. He is presently the director of the Falk Institute for Mental Health Research, Jerusalem, Israel.
